# Magnetically induced forward scattering at visible wavelengths in silicon nanosphere oligomers

**DOI:** 10.1038/ncomms8042

**Published:** 2015-05-05

**Authors:** J. H. Yan, P. Liu, Z. Y. Lin, H. Wang, H. J. Chen, C. X. Wang, G. W. Yang

**Affiliations:** 1 State Key Laboratory of Optoelectronic Materials and Technologies, Nanotechnology Research Center, School of Physics & Engineering, Sun Yat-sen University, Guangdong 510275, China

## Abstract

Electromagnetically induced transparency is a type of quantum interference that induces near-zero reflection and near-perfect transmission. As a classical analogy, metal nanostructure plasmonic ‘molecules' produce plasmon-induced transparency conventionally. Herein, an electromagnetically induced transparency interaction is demonstrated in silicon nanosphere oligomers, wherein the strong magnetic resonance couples with the electric gap mode effectively to markedly suppress reflection. As a result, a narrow-band transparency window created at visible wavelengths, called magnetically induced transparency, is easily realized in nearly touching silicon nanospheres, exhibiting low dependence on the number of spheres and aggregate states compared with plasmon induced transparency. A hybridization mechanism between magnetic and electric modes is proposed to pursue the physical origin, which is crucial to build all-dielectric metamaterials. Remarkably, magnetic induced transparency effect exhibiting near-zero reflection and near-perfect transmission causes light to propagate with no extra phase change. This makes silicon nanosphere oligomers promising as a unit cell in epsilon-near-zero metamaterials.

## Abstract (web summary)

A weak and narrow electric dipole has limited the use of silicon nanospheres in nanophotonic applications requiring strong interaction between electric and magnetic modes. Here, Yan *et al.* demonstrate effective coupling between the magnetic resonance and the electric gap mode in nearly touching silicon nanospheres.

## Graphical abstract

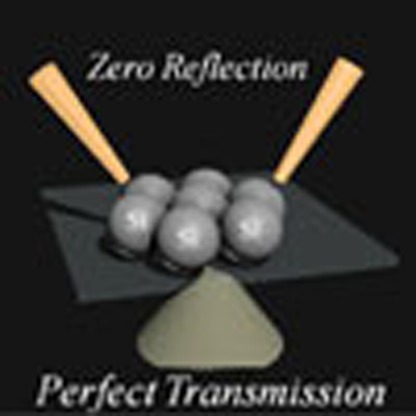

Silicon nanospheres are excellent scatterers possessing directional scattering and low-loss properties in the visible region, making them superior to noble metals in some applications such as directional nanoantennas^1^,^2^ and visible light communication^3^. The strong magnetic response with low loss in silicon nanospheres is crucial for their nanophotonics applications^4^,^5^,^6^,^7^. Although the magnetic dipole resonance is quite strong in a silicon nanosphere, the electric dipole and higher-order contributions are relatively weak and narrow, so these electric modes cannot couple effectively with the magnetic mode. However, some optical phenomena such as Fano resonance^8^,^9^, negative refraction^10^ and cloaking^11^ require a strong interaction between the electric and magnetic modes. Fortunately, a strong interaction such as this can be observed in nearly touching silicon oligomers, wherein the junction between the two proximal spheres can generate a strong and broad electric field enhancement^12^ that can interfere with the magnetic mode. It should be noted that previous studies on detached silicon oligomers fabricated via electron-beam lithography have not realized a strong electromagnetic interaction^13^,^14^,^15^, and have thus exhibited no directional feature^16^. Owing to their unique electromagnetic interaction, silicon nanosphere oligomers exhibit novel mechanisms in some classical analogous quantum effects, and especially in electromagnetically induced transparency (EIT), which is a quantum interference effect in three-level atomic systems that eliminates the absorption at the resonance frequency and gives rise to a narrow transparency window^17^,^18^. Combining EIT with plasmonic structures, the destructive interference between overlapped narrow and broad plasmonic modes creates plasmon-induced transparency (PIT).^19^,^20^,^21^,^22^,^23^ Although the EIT-like features of PIT have important applications in ultrasmall sensors and optical metamaterials^19^,^20^, the relative large ohmic loss in the metals confines the applications of PIT to the visible wavelengths. Recent studies replacing metals with all-dielectric materials have yielded metamaterials that exhibit EIT^24^, negative refraction^25^, artificial magnetic conduction^26^ and the production of a zero-index metamaterial^27^. However, these all-dielectric nanostructures either cannot function in the visible region or are fabricated by complex top-down methods such as electron-beam lithography.

To overcome these issues, we report the first experimental demonstration of EIT-like phenomena in the visible region based on arbitrarily aggregated silicon nanospheres. By a simple and effective self-assembly method, we fabricate arbitrary silicon nanosphere oligomers in the nearly touching regime between two spheres. According to the Mie theory and dipole–dipole interaction^12^, electric dipoles in the individual spheres combine to a broad electric response with strong electric field enhancement in the gap, and magnetic dipoles combine to a narrow hybrid magnetic response in the oligomers. This broad electric gap mode hybridizes with the narrow magnetic resonances, which leads to transmission enhancement and antireflection. Therefore, we call this phenomenon magnetically induced transparency (MIT). Note that the MIT mechanism based on electromagnetic interaction is distinctly different from the mechanism of PIT in plasmonic nanostructures. In addition, unlike metal plasmonic oligomers^28^,^29^,^30^, the characteristic spectra of silicon nanosphere oligomers are less dependent on the number of particles, the states of aggregation and even the size of the distributions. Accordingly, EIT can be much more easily realized in all-dielectric nanospheres, which is significant for promising applications. More importantly, the MIT with near-zero reflection and near-perfect transmission causes light to propagate through with no extra phase change at a particular wavelength. Thus, we consider that silicon nanosphere oligomers are an important all-dielectric nanostructure that can perform as a unit cell to build MIT-based epsilon-near-zero (ENZ)^27^,^31^,^32^ metamaterials.

## Results

### MIT effect in silicon nanosphere trimers

**Figure 1:**
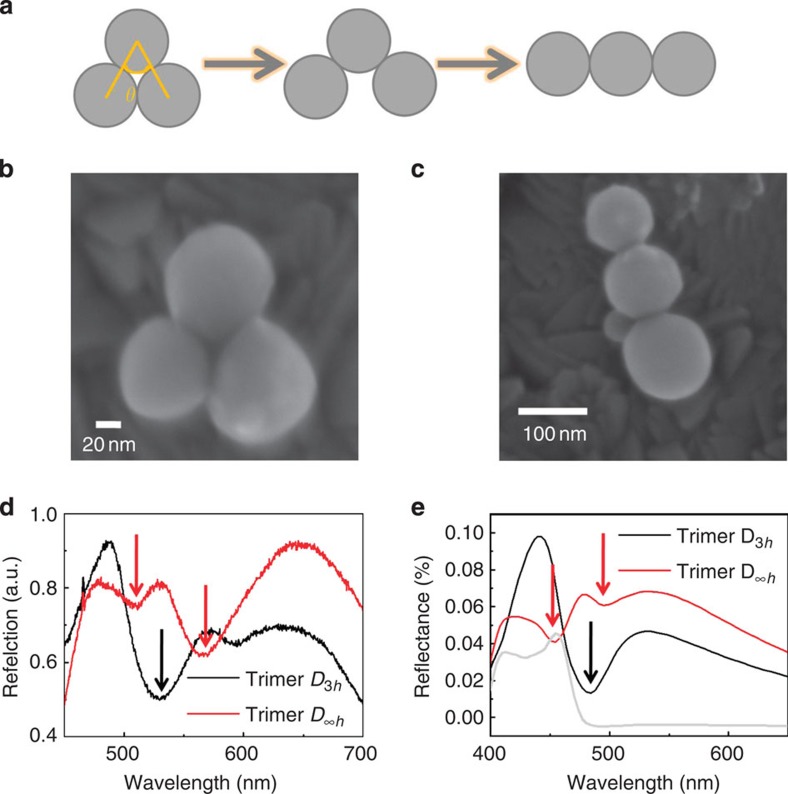
The antireflection phenomenon in silicon nanosphere trimers with different aggregate states. (**a**) Schematic diagram of different trimers depending on the vertex angle *θ*. When *θ*=60°, trimer belongs to the *D*_3*h*_ symmetry group. When *θ*=180°, trimer belongs to the *D*_∞*h*_ symmetry group. (**b**) SEM image of a typical *D*_3*h*_ trimer. (**c**) SEM image of a *D*_∞*h*_ trimer. (**d**) The measured dark-field reflection spectra of *D*_3*h*_ (black curve) and *D*_∞*h*_ trimers (red curve). Three arrows show the locations of three dips caused by the electro-magnetic interactions. (**e**) The simulated dark-field reflection spectra of *D*_3*h*_ (black curve) and *D*_∞*h*_ trimers (red curve). Similarly, the arrows show the locations of three dips caused by the electro-magnetic interactions. The particle sizes are set to 100 nm, and the grey curve shows the magnetic and electric dipole resonances of single 100 nm silicon sphere.

Femtosecond laser ablation in liquid^33^ is used to prepare silicon nanospheres with an average size of about 100 nm. There are two reasons for isolated silicon nanospheres to form oligomers. First, the heating effect during the laser ablation melts the sphere surface and causes the spheres to adhere to each other. Second, the self-assembly process along with the evaporation of water generates a capillary force that holds several spheres together. To investigate the interaction of more complex silicon nanosphere oligomers, first we must understand the mechanism occurring in a trimer. Different types of silicon trimers are shown in Fig. 1a. Increasing the vertex angle can decrease the interaction among the three spheres, so we choose two typical trimers for dark-field reflection spectra comparison. Two trimer species belonging to the *D*_3*h*_ symmetry group (equilateral triangle) and the *D*_∞*h*_ symmetry are shown in Fig. 1b,c, respectively, and the diameters of each sphere are measured using scanning electron microscope (SEM) images via multiple measurements. The diameters in the *D*_3*h*_ trimer are 82, 98 and 98 nm, while the diameters in *D*_∞*h*_ trimers are 90, 112 and 124 nm and, although size deviations exist, the discrepancy is minimal. In addition, smaller spheres with a relatively large deviation (e.g., 82-nm sphere in *D*_3*h*_ trimers and 90-nm sphere in *D*_∞*h*_ trimers) contribute much less to the reflection spectra, which is demonstrated later. Decreased dependence on size distribution is an advantage for fabricating oligomer structures and for easily attaining the MIT effect.

In Fig. 1d, the reflection spectra of the *D*_3*h*_ and *D*_∞*h*_ trimers obtained by a dark-field microscope are presented (see details in Methods), and the spectral shapes can be seen to be very different. Only one deep magnetically induced dip can be observed in the *D*_3*h*_ trimer, and the other shallow dip at 600 nm is not produced by the strong magnetic resonance in the silicon spheres because it is located far away from the magnetic dipole resonance of a single silicon sphere (see grey curve in Fig. 1e). In the *D*_∞*h*_ trimer, however, two shallow dips can be observed because the symmetry in the *D*_∞*h*_ trimer is much lower than that of the *D*_3*h*_ trimer, which possesses the highest symmetry. Therefore, the three spheres in the *D*_∞*h*_ trimer cannot reach the maximum magnetic response simultaneously, so they interact with the electric mode individually and cause the two shallow dips. To verify these experimental observations, numerical simulations are carried out using the finite-difference time-domain (FDTD) method, and the total-field scattered-field source and a backward plane detector are set to simulate the dark-field reflection in the experiments more accurately (see Methods). First, the simulation is conducted based on the sizes obtained from multiple measurements, as shown in Supplementary Fig. 1. The simulated results accord with the experimental results well, though some discrepancies in the peak or valley wavelengths may arise from measuring errors or defects in the silicon sphere that influence the magnetic resonant wavelengths. Further, to investigate the MIT effect without loss of generality, the diameters of the silicon spheres in the simulation are set to 100 nm to make analysis simple and clear. The simulated spectra in Fig. 1e are similar to the experimental results in Fig. 1d, which demonstrates that the MIT effect has little dependence on size distribution. A dominant antireflection dip in the *D*_3*h*_ trimer and two shallow dips in the *D*_∞*h*_ trimer can also be clearly seen, so we can conclude that the antireflection effect can be realized in all silicon nanosphere trimers with the degree of antireflection depending on the aggregate state.

### Theoretical analysis on the electromagnetic interaction

**Figure 2:**
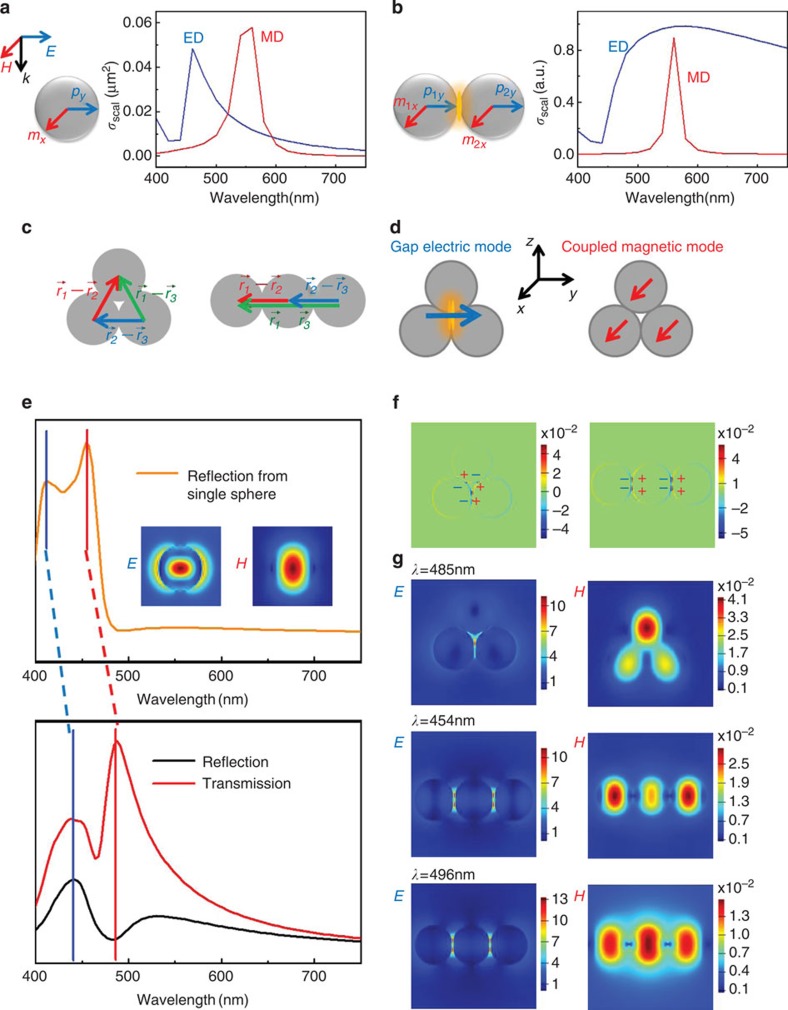
Mechanism of MIT. (**a**) The scattering cross-sections contributed by magnetic dipole (MD) and electric dipole (ED) in an individual silicon sphere with diameter of 130 nm. (**b**) The scattering cross-sections contributed by magnetic dipole and electric dipole in a silicon nanosphere homodimer with diameters of 130 nm. (**c**) The schematic shows the difference between two kinds of trimer when calculating the interaction between each of two spheres. (**d**) The schematic describes two new modes: the gap electric mode and the coupled magnetic mode. (**e**) The hybridization process in a typical *D*_3*h*_ trimer with diameter of 100 nm. The blue bar reveals the variation from electric dipole mode in the individual sphere to the gap electric mode. The red bar reveals the variation from magnetic dipole mode to the coupled magnetic mode. The insets in the individual sphere's spectrum are field distributions at resonance wavelengths. Moreover, the difference between transmission and reflection spectra indicates the directivity caused by the electro-magnetic interaction. (**f**) The charge density distributions of *D*_3*h*_ and *D*_∞*h*_ trimers. (**g**) The electric field distribution of the gap mode and the magnetic field distribution of the *D*_3*h*_ trimer at 485 nm where the antireflection dip happens. The broad electric gap mode and the maximum magnetic responses of the *D*_∞*h*_ trimer at 454 and 496 nm.

To understand how the structure influences the reflection spectra, we must understand how the electric and magnetic modes change as the structure changes from a single silicon sphere to a silicon oligomer. For an optical nanoantenna, the scattered fields can be represented as the superposition of the fields created by a set of multipole modes^34^. For a silicon sphere with a diameter <150 nm, however, higher-order resonances such as quadrupole responses are much weaker than dipole responses^7^, so it is sufficient to consider only the dipole–dipole interaction. The scattering cross-section contributed by the electric dipole and the magnetic dipole can be described as^35^


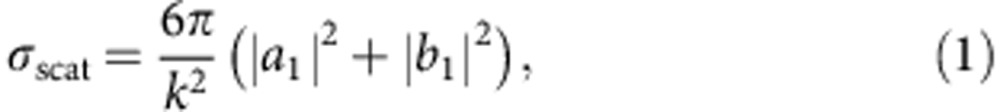


where *k* is the wave vector and *a*_1_ and *b*_1_ are the Mie coefficients for the electric and magnetic dipoles, respectively. The calculated spectra in Fig. 2a indicate that the magnetic and electric dipole resonances are narrow and detached and cannot couple with each other effectively. When combining two silicon spheres, the modes change markedly and reveal interesting phenomena. Under the excitation polarized along the dimer axis (*y*-axis), the induced magnetic dipole, **m**_1*x*_ and **m**_2*x*_, and the induced electric dipole, **p**_1*y*_ and **p**_2*y*_, can be generated as (Fig. 2b)^12^


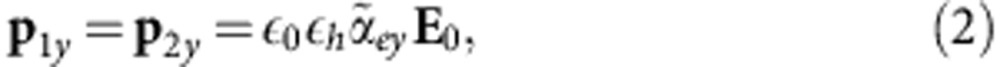



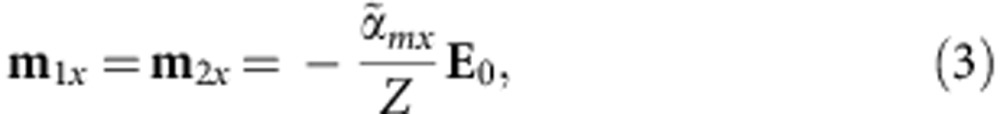


where 
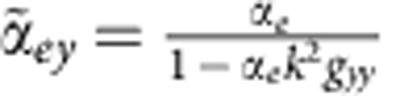
 and 
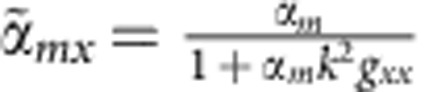
 can be defined as the equivalent polarizabilities; *ɛ*_0_ and *ɛ*_*h*_ are the vacuum and relative permittivity, respectively; *Z*=(*μ*_0_*μ*_*h*_/(*ɛ*_0_*ɛ*_*h*_))^1/2^ is the vacuum impedance; *k* is the wavenumber; *E*_0_ represents the incident electric field; *g*_*xx*_ and *g*_*yy*_ are scalar Green's functions related to the distance between two spheres; and *α*_*e*_=(6*πi*/*k*^3^)*a*_1_ and *α*_*m*_=(6*πi*/*k*^3^)*b*_1_ are the electric and magnetic polarizabilities, respectively, in a single sphere and are related to the Mie coefficients *a*_1_ and *b*_1_. The expressions of the equivalent polarizabilities 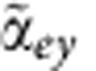 and 
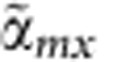
, which are similar to the expressions in a single sphere, represent the new electric gap mode and a hybrid magnetic dipole mode. By substituting 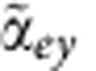 and 
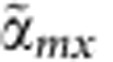
 into equation (1), the scattering cross-section of a homodimer nanostructure contributed by each coefficient can be calculated, as shown in Fig. 2b. The electric dipole mode becomes broader while the magnetic dipole mode becomes narrower, so the modes can overlap and couple with each other effectively. This mode variation understanding can be easily translated to the trimer structures, but the mode variations in *D*_3*h*_ and *D*_∞*h*_ trimers differ from each other because of the different distances between each pair of spheres. Under the excitation polarized along the *y*-axis, the induced magnetic dipoles can be described as


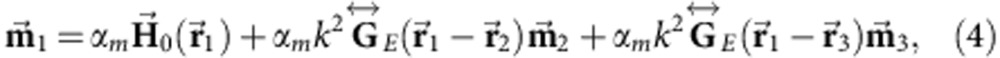


where 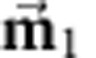, 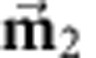 and 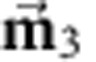 represent the induced magnetic dipoles in each of the three spheres; 
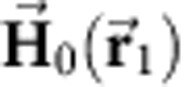
 is the incident magnetic field; and the dyadic Green's functions 
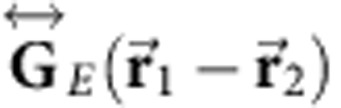
 and 
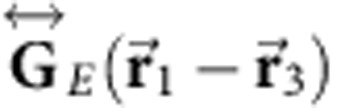
, respectively, represent the effects of spheres Nos. 2 and 3 upon sphere No. 1. In the *D*_3*h*_ trimer, the relations 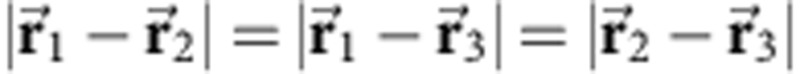 mean that the magnetic responses of each sphere are uniform (Fig. 2c), while in the *D*_∞*h*_ trimer, the relations 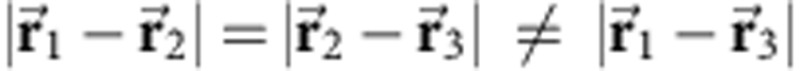 mean that the central sphere's magnetic response is discrepant with the two side spheres (Fig. 2c). This can explain why there is one antireflection dip originating from the *D*_3*h*_ trimer and two dips from the *D*_∞*h*_ trimer.

As discussed above, the coupling of electric dipoles in individual spheres leads to the generation of a gap electric mode that is broad and bright, and the coupling of magnetic dipoles produces a hybrid magnetic mode that is narrow and dark (Fig. 2d). The interaction mechanism and the MIT effect can be further understood by the hybridization model^36^. Taking the *D*_3*h*_ trimer for an example, Fig. 2e illustrates how the resonance modes in a single sphere hybridize to form new electric and magnetic dipole modes. The overlapping of the energy levels of the electric dipole modes from three spheres causes the levels to hybridize strongly with each other and produce a broad and bright background in the spectra (blue dotted line in Fig. 2e). The electric field distributions of a single sphere (inset in Fig. 2e) and a silicon trimer (Fig. 2g) describe the change from the typical electric dipole mode to the gap electric mode, respectively. In contrast, the hybridization of three magnetic dipoles forms a new magnetic dipole mode at *λ*=485 nm in Fig. 2e. The circuit displacement currents caused by the ‘narrow' coupled magnetic mode is near the surface, so the currents can interfere with the ‘bright' gap electric mode effectively in a narrow spectral range. This unique electromagnetic interaction will generate Kerker-type directional scattering, which is totally different from the electric dipole–dipole interaction in plasmonic structures. This directivity is given by ref. 16


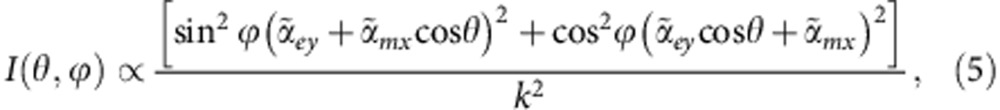


where *θ* is the scattering angle and *ϕ* is the angle between the incident field and the scattering plane. When 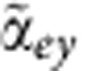 and 
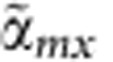
 have the same value, near-zero reflection (*θ*=0°) and near-perfect transmission (*θ*=180°) can be achieved at the same time (*λ*=485 nm in Fig. 2e).

To understand the difference between the two kinds of trimers, the charge density distributions in Fig. 2f can provide an intuitive explanation. In the *D*_3*h*_ trimer, under the excitation polarized along the *y*-axis, charges distribute on the surface and especially localize around the gap in the centre. These localized surface charges have a Coulomb interaction^37^ with the displacement current inside the spheres, where the charge centralized area has an identical effect on the displacement current of all three spheres because of the equal and ultrasmall intervals, so the coupling in the *D*_3*h*_ trimer causes the three spheres to reach the maximum magnetic response at the same time. The situation in the *D*_∞*h*_ trimer is different (right in Fig. 2f), however, because the charge distributions are more dispersed and located at both junctions, which causes the Coulomb interaction with the centre sphere's magnetic dipole to be different from the interaction with side spheres' magnetic dipoles. The high-density positive and negative charges on both sides of the centre sphere generate a strong static electric field and weaken the magnetic resonance energy, which is why the magnetic responses of the centre sphere and side spheres are not synchronized and the antireflection dip caused by the centre sphere is more red-shifted (Fig. 1e). To confirm this analysis, the near-field distributions are calculated by the FDTD method at three antireflection dips (Fig. 2g), namely, the antireflection dip of the *D*_3*h*_ trimer located at 485 nm and the two dips of the *D*_∞*h*_ trimer located at 454 and 496 nm. The electric fields in these two kinds of trimers have more than 10 times enhancement, which indicates that the gap mode is strong and broad. Compared with the electric gap mode, the magnetic dipole moments are more changeful. In the *D*_3*h*_ trimer, all three spheres reach the maximum magnetic response synchronously at 485 nm, but in the *D*_∞*h*_ trimer, the largest magnetic response of the two side spheres is located at 454 nm while the centre sphere reaches the maximum magnetic resonance at 496 nm. Although the three spheres in the *D*_3*h*_ trimer do not possess equal magnetic dipole moments, the simulations show that they do reach the maximum at the same time by calculating the precise magnetic field enhancement at the centre of each sphere (Supplementary Fig. 2). The top sphere has the largest dipole moment because the high-density surface charges near the gap have a sign reversal (Fig. 2f), so the displacement current in the top sphere cannot be weakened from the cancellation of positive and negative surface charges.

### MIT effect in silicon nanosphere tetramers

**Figure 3:**
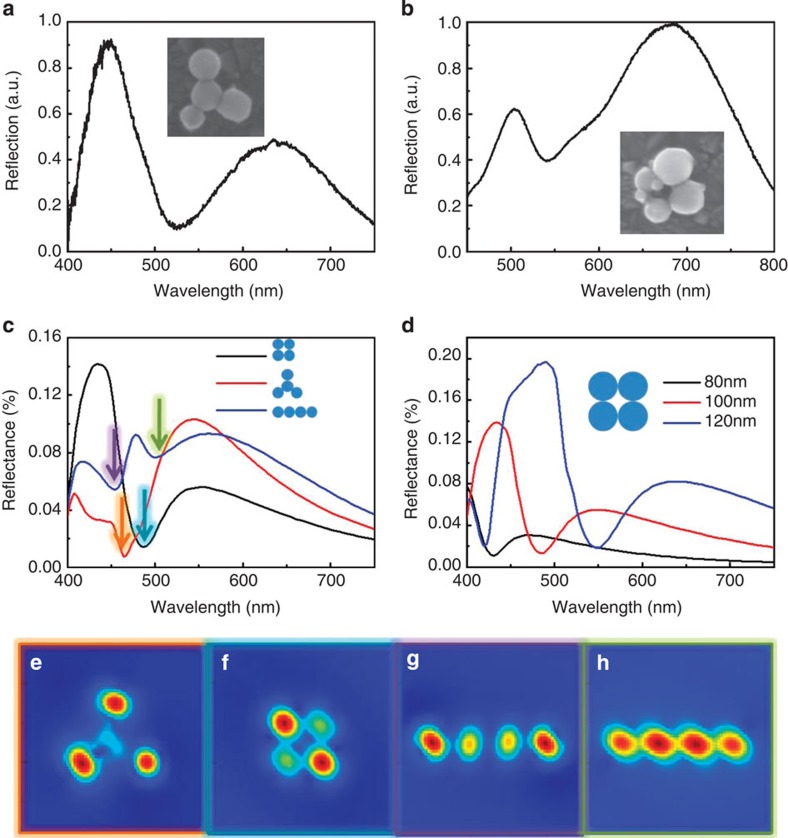
MIT in silicon nanosphere tetramers. (**a**) The dark field reflection spectrum of a Y-type tetramer (*d*=100, 99, 98 and 72 nm). (**b**) The dark field reflection spectrum of a rectangular tetramer (*d*=136, 126, 92 and 84 nm). The insets in (**a**) and (**b**) show SEM images of two kinds of tetramer. (**c**) The simulated reflectance spectra of three kinds of tetramer. The diameters are set to 100 nm. (**d**) Reflectance spectra of rectangular tetramer varied with sphere diameter. (**e**–**h**) The magnetic field distributions in boxes with different colours corresponding to the dips marked with specific arrows in (**c**).

On the basis of the comparison of these two kinds of trimers, the mechanism for MIT generation becomes clear and we can thereby predict that silicon nanosphere oligomers with high symmetry and a close-packed structure can effectively produce MIT. Essentially, the synchronous and strong magnetic response interacting with the localized gap electric mode will cause a dominant antireflection dip to realize MIT. The experimental spectra in Fig. 3a,b verify these predictions, wherein a Y-type tetramer is seen to exhibit a near-zero antireflection dip at 524 nm and a rectangular tetramer attains MIT at 540 nm, respectively. The simulated results in Fig. 3c are consistent with these experimental results, wherein the Y-type and rectangular tetramers exhibit only one antireflection dip, and the difference between the two dips is attributed to the differing electromagnetic interactions discussed in the trimer situation. Similarly, the tetramer with a linear shape has two dips comparable to the *D*_∞*h*_ trimer. As we know, the electric gap modes are quite broad, so the interaction between electric and magnetic modes mainly depends on the variation of the coupled magnetic dipole modes. To verify the antireflective properties in different aggregate states, the magnetic field distributions are presented in Fig. 3e–h. In the Y-type tetramer, the dominant dip is generated by the strong magnetic responses from three ambient silicon spheres (Fig. 3e). Similarly, in the rectangular tetramer, the high symmetry causes the four spheres to reach the strongest magnetic resonances synchronously. The strong magnetic response hybridizes with the gap electric mode effectively and gives rise to MIT (Fig. 3f). In the linear shape tetramer, however, the middle two spheres and the side two spheres have different coupling situations and reach the strong magnetic resonances asynchronously, as shown in Fig. 3g,h. In addition, Fig. 3d explores the influence of particle sizes on the MIT effect where, taking the rectangular tetramer as an example, we can see that the antireflection dip red-shifts and the intensity of the peaks is enhanced when the sphere diameters increase. This phenomenon agrees with the scattering property of a single silicon sphere, as shown in Supplementary Fig. 3, which can be explained using the Mie theory.

This result indicates that silicon spheres with larger sizes dominate the spectral response in an oligomer and, as shown in Supplementary Fig. 4, when some of the sphere diameters are decreased in an oligomer, their influence on the MIT effect can be ignored. First, the diameter (*d*) of one sphere in a rectangular tetramer is reduced from *d*=120 to 80 nm (Supplementary Fig. 4a), but little influence of this reduction is seen on the antireflection dips around 550 nm (Supplementary Fig. 4c). Second, the diameters of two spheres in a rectangular tetramer are reduced (Supplementary Fig. 4b), and there is still little influence seen on the dips around 550 nm. Smaller spheres whose magnetic dipole resonances are much weaker and far away from the antireflection dip in the spectrum cannot couple with the dominant magnetic response, which makes the MIT effect less dependent on particle sizes and allows the existence of a size distribution in the oligomer, which is beneficial for future applications. The MIT effects of tetramers in Fig. 3a (*d*=100, 99, 98 and 72 nm) and Fig. 3b (*d*=136, 126, 92 and 84 nm) verify this conclusion well via both experiments (Fig. 3a,b) and simulation (Supplementary Fig. 5).

## Discussion

**Figure 4:**
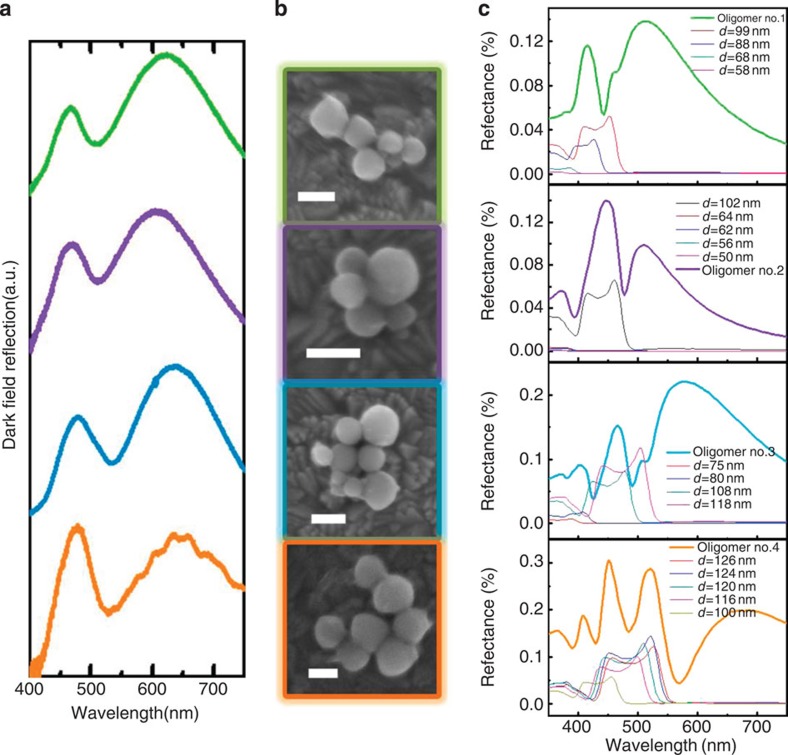
MIT in arbitrary silicon nanosphere oligomers. (**a**) The measured dark field reflection spectra of four different oligomers. The antireflection dips are located between 500 and 550 nm. (**b**) SEM images of the corresponding oligomers. Scale bar, 100 nm. (**c**) The simulated reflectance spectra (thick curves) of four oligomers and the scattering spectra of the individual sphere in each oligomers are presented by thin curves as a comparison.

Diminished control of the oligomer structure and size may be a disadvantage of the self-assembly method compared with top-down methods but, fortunately, the universal electromagnetic hybridization mechanism guarantees the generation of MIT regardless of the aggregate states and particle sizes. In addition, evident MIT can only be generated in nearly touching self-assembled oligomers. The dark-field reflected spectra of four arbitrary silicon nanosphere oligomers are shown in Fig. 4, where a distinct MIT effect can be clearly observed between 500 and 550 nm in all four spectra (Fig. 4a). Despite the differences existing in the degree of transparency and the intensity of two peaks in the different oligomers, the MIT effects are almost identical in these oligomers. On the basis of the SEM images of four different oligomers in Fig. 4b and the simulated spectra in Fig. 4c, we can explain further why the MIT effect exhibits little dependence on particle size. For the pentamer in the green box in Fig. 4b, the sphere with *d*=99 nm on the left and the two spheres with *d*=88 nm possess strong magnetic responses that lead to the antireflection effect, while the remaining smaller spheres act as supplements to generate the electric gap mode. The simulated result in Fig. 4c accords well with these experimental results, and the scattering spectra of each individual sphere indicate that the contributions to the antireflection dip from smaller spheres are negligible. The antireflection dips caused by the spheres with *d*=99 and 88 nm are in close proximity in the spectrum (Fig. 4c), so they broaden and hybridize to create one MIT dip in the experimental spectrum. Similarly, the pentamer in the purple box in Fig. 4b is a 102-nm-diameter sphere surrounded by four much smaller spheres. The broad gap electric mode can be produced, but only the dominant magnetic response in the *d*=102 nm sphere hybridizes with the gap mode and causes an antireflection dip around 500 nm, as shown in Fig. 4a,c (purple curve). The oligomer in the blue box in Fig. 4b is similar to a rectangular hexamer, so we neglect the smaller spheres and only consider the two spheres with *d*=108 and 118 nm. These two spheres interact with the gap mode and generate MIT, and the simulated result accords well with the experimental result, as shown in Fig. 4a,c (blue curve). Finally, the heptamer in the orange box in Fig. 4b can also produce an antireflection dip at 533 nm based on the strong electromagnetic interaction (Fig. 4a), wherein the magnetic resonances of the four spheres with *d*=126, 124, 120 and 116 nm couple together and dominate the MIT effect. Although three 100-nm-diameter spheres can also generate an antireflection dip, as shown in Fig. 4c (orange curve), the relatively narrow dip disappears in the experimental spectrum because of spectral broadening. Some discrepancies in the MIT wavelengths between the simulated and experimental results may be caused by defects in silicon spheres that affect the magnetic response and the different gap distances or relative altitudes that influence the gap mode. Meanwhile, the simulated results of several regular oligomers (Supplementary Fig. 6) also exhibit the MIT effect regardless of their aggregate states and number of spheres.

**Figure 5:**
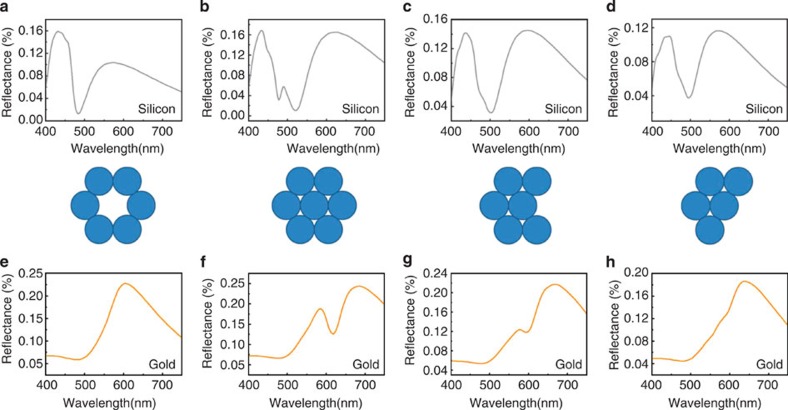
Comparison between silicon oligomers and plasmonic oligomers. (**a**–**d**) The dark-field reflectance spectra of four different silicon oligomers. They are ring-like hexamer, heptamer, hexamer and pentamer, respectively (shown in the middle). (**e**–**h**) The dark-field reflectance spectra of four different gold oligomers.

The comparison between silicon and gold nanosphere oligomers in Fig. 5 gives an intuitive understanding of the difference between electromagnetic hybridization and plasmonic hybridization. For a silicon nanosphere hexamer (ring shape), the gap electric mode can be generated in the junctions of each pair of spheres, which interferes with the magnetic response destructively in the backward direction and causes the MIT effect at 485 nm (Fig. 5a). After adding one sphere in the center of the hexamer to form a heptamer, the different hybridization situation causes the dip wavelength to shift a little but the obvious MIT effect remains unchanged at around 500 nm (Fig. 5b). Further, when removing one or two side spheres from the heptamer structure, the MIT effect remains unchanged (Fig. 5c,d). For a gold hexamer (ring shape), however, the six spheres couple in an in-phase electric dipole mode to create a broad spectrum (Fig. 5e). After transforming the hexamer to a heptamer structure, a shallow dip arises (Fig. 5f) because of the Fano resonance from the hybridization of the plasmons in the central sphere and side spheres. This Fano dip is sensitive to the structural symmetry, so the Fano dip disappears and the whole spectrum becomes broader when the side spheres are removed, as shown in Fig. 5g,h. The absence of several side spheres weakens the super-radiant mode, and thereby suppresses the Fano resonance. Summarizing the discussion above, we can conclude that silicon nanosphere oligomers can easily give rise to a distinct MIT effect as long as the oligomer spheres are relatively close-packed to produce the electric gap mode and relatively well distributed in size to guarantee that several large spheres can generate the strong magnetic response. The large tolerance of the size and structure in all-dielectric oligomers is a unique change from conventional metal plasmonic nanostructures. In addition, silicon nanospheres possess less intrinsic losses (Im *ɛ*=1.3–0.4 in the spectral range of 400 nm <*λ*<550 nm) than gold spheres (Im *ɛ*=5.8–1.8 in the spectral range of 400 nm <*λ*<550 nm), making silicon nanospheres superior to assembly as nanoantennas or metamaterials. The reflectance and transmittivity spectra of silicon and gold heptamers (Supplementary Fig. 7) indicate that the near-perfect transmission peak of the silicon oligomer is much stronger than the transmission from the gold oligomer because of the low-loss property and directional electromagnetic interaction of the silicon oligomer.

As discussed above, when new magnetic and electric dipoles meet certain relationship requirements, near-zero backward scattering and near-perfect forward scattering will occur. At a specific wavelength, the structure appears transparent, or ‘invisible', and the incident waves experience no extra phase change when passing through. Thus, we can predict that this unique MIT structure can act as an ENZ metamaterial at visible wavelengths. A close-packed 100-nm sphere diameter silicon nanosphere array is simulated, as shown in Supplementary Fig. 8a. From the reflection and transmission spectra in Supplementary Fig. 8b, we can see a near-zero dip in the reflection spectrum at 480 nm corresponding to the perfect transmission peak (red curve). The phase images in Supplementary Fig. 8c,d show the phase change without and with the silicon nanosphere array, respectively. Without the nanosphere array (Supplementary Fig. 8c), the phase change indicates the propagation of plane wave; but when the plane wave interferes with the silicon nanosphere array at the MIT wavelength (Supplementary Fig. 8d), the phase in the backward direction remains unchanged, leading to near-zero reflection, and in the forward direction the silicon nanosphere array realizes the spatial modulation and recovers the plane wave features in the far field. As a whole, the incident plane wave experiences no extra phase change when passing through.

In summary, we experimentally demonstrate the MIT effect in silicon nanosphere trimers, tetramers and other arbitrary oligomers. By investigating the dipole–dipole interaction in detail, we are able to analyse the electromagnetic interactions in different oligomers, wherein the hybridization between the gap electric mode and the coupled magnetic mode can induce the universal electromagnetic interaction to create antireflection at a specific wavelength. Compared with PIT, MIT can be much more easily generated regardless of the particle size, particle number and aggregate state of the oligomers. These findings demonstrate that silicon nanosphere oligomers possessing MIT have promising applications in ultrasmall sensors and optical metamaterials, and especially in ENZ metamaterials.

## Methods

### Fabrication of silicon nanospheres oligomers

Silicon nanospheres with smooth and clean surfaces were prepared by femtosecond laser ablation of a silicon wafer in deionized water using a Legend Elite Series ultrafast laser (Coherent) with a pulse duration of 35 fs and a single-pulse energy of 4 mJ. After laser ablation, the silicon colloid was suspended in solution and one drop of the solution was transferred to a piece of clean indium tin oxide (ITO) glass. During the evaporation process, big droplets split into small droplets containing fewer silicon spheres and, when these droplets dried up, multiple silicon spheres were self-assembled as dimers, trimers or other oligomers via capillary forces. In addition, the sintering effect during laser ablation also assisted in the aggregation.

### Dark-field reflection measurements

The dark-field reflection of silicon nanosphere oligomers, also called backward scattering spectra, were collected using a dark-field optical microscope (Olympus BX51) integrated with a quartz-tungsten-halogen lamp, a monochromator (Acton SpectraPro, 2300i) and a charge-coupled device (CCD) camera (Princeton Instruments, Pixis 400BR_eXcelon). During the measurements, the camera was thermoelectrically cooled to −70 °C. The oblique incident white light was illuminated with a 53° incident angle on the oligomer, and the scattered light was collected by a dark-field objective on top (LMPLFLN100XBD, numerical aperture=0.80).

### Numerical calculations

The reflection spectra, charge distribution and near-field distributions were calculated using the FDTD method (FDTD Solutions 8.6.0, Lumerical Solutions). The normal incident total-field scattered-field plane wave at visible wavelengths (300–900 nm) combined with a planar detector was used to effectively simulate the dark-field reflection found in the experiment. The oligomer nanostructures were illuminated by a plane wave with both unit s-polarized and p-polarized components to simulate the unpolarized source in the experiment. A mesh size of 2 nm for the illuminated region was used, and the dielectric function of silicon was obtained from Palik.^38^ In the simulations, we calculated the spectra in free space and ignored the substrate because the ITO glass substrate will have little effect on the backward scattering spectra.

## Author contributions

J.H.Y, P.L, Z.Y.L. and H.W.: experimental work. H.J.C. and C.X.W: data analysis. G.W.Y: project planning, data analysis.

## Additional information

**How to cite this article:** Yan, J. H. *et al.* Magnetically induced forward scattering at visible wavelengths in silicon nanosphere oligomers. *Nat. Commun.* 6:7042 doi: 10.1038/ncomms8042 (2015).

## Supplementary Material

Supplementary Information Supplementary Figures 1-8
