# Supplementary material for: Magnetically induced forward scattering at visible wavelengths in silicon nanosphere oligomers
Source: Nat Commun. 2015 May 5;6:7042. doi: 10.1038/ncomms8042 (PMC4432586; doi:10.1038/ncomms8042)
Supplement: Supplementary Information — Supplementary Figures 1-8 [file ncomms8042-s1.pdf]

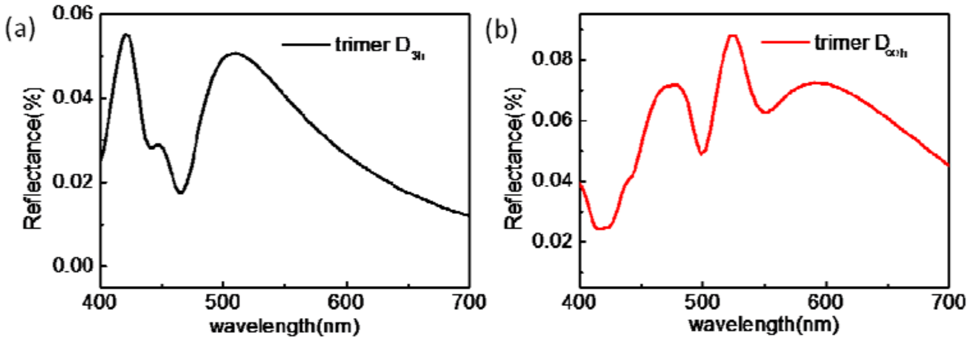

Supplementary Figure 1. The simulated spectra of two kinds of silicon nanosphere trimer. (a) The simulated reflectance spectrum of the D_{3h} trimer whose diameters are set to 98 nm, 98 nm and 82 nm according to the SEM images. (b) The simulated reflectance spectrum of the $D_{\infty h}$ trimer whose diameters are set to 124 nm, 112 nm and 90 nm according to the SEM images.

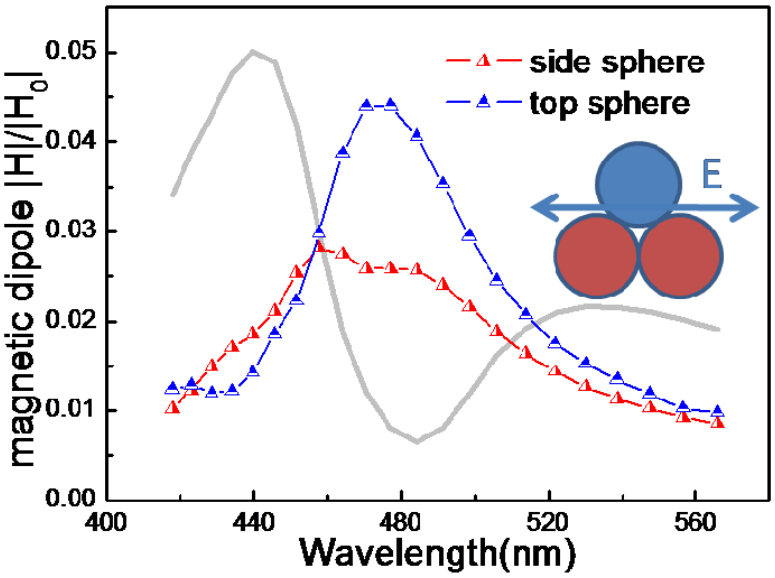

Supplementary Figure 2. The magnetic dipole moments variation of three spheres in D_{3h} **trimer.** The inset shows the relationship between the polarization direction and three spheres. Blue line represents the magnetic response of top sphere, red line represents the magnetic response of two side spheres, and gray line shows the simulated reflection spectra. The antireflection dip corresponds to the maximum magnetic responses of all three sphere. The diameter of all three spheres is 100nm.

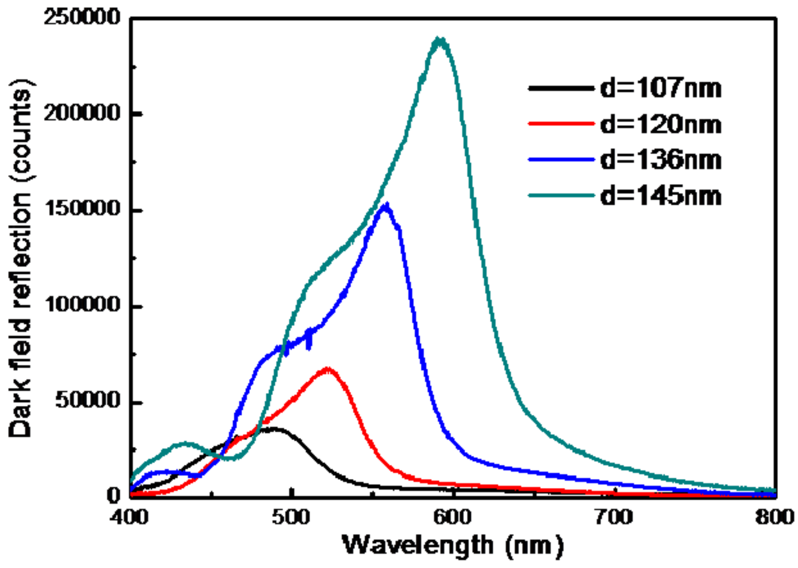

Supplementary Figure 3. The scattering property of single silicon nanospheres with different sizes. The dark field reflection spectra of silicon spheres with the diameters of 107, 120, 136 and 145 nm. The intensity increases and the peak red shifts when increasing the diameter.

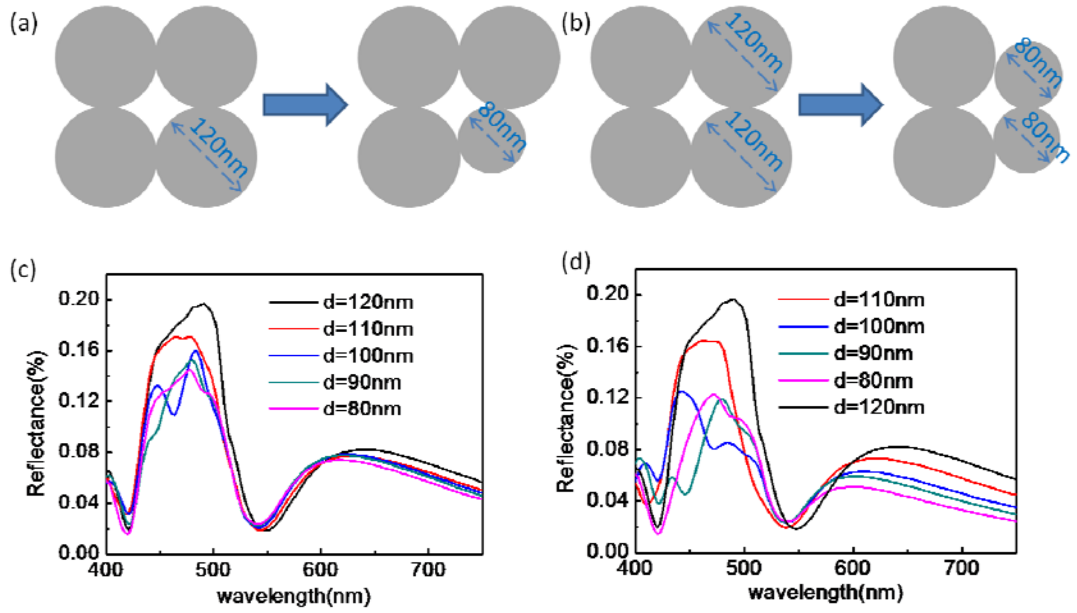

Supplementary Figure 4. The influence of size differences on the MIT effect in oligomers. (a) The schematic shows decreasing one sphere's diameter in tetramer from 120 nm to 80 nm gradually. (b) The schematic shows decreasing two sphere's diameter in tetramer from 120 nm to 80 nm gradually. (c) The corresponding reflectance spectra varied with the diameter of one decreasing sphere. (d) The corresponding reflectance spectra varied with the diameter of two decreasing spheres.

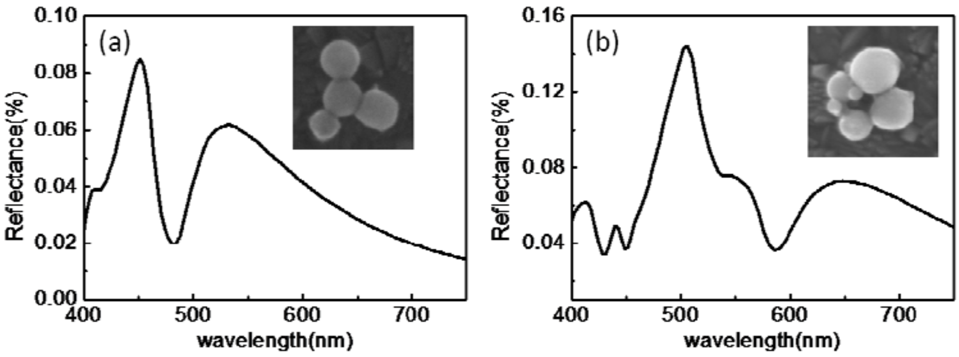

Supplementary Figure 5. The simulated spectra of two kinds of silicon nanosphere tetramer. (a) The simulated reflectance spectrum of the Y-type tetramer whose diameters are set to 100, 99, 98 and 72 nm according to the SEM image (inset). (b) The simulated reflectance spectrum of the rectangular tetramer whose diameters are set to 136, 126, 92 and 84 nm according to the SEM images (inset).

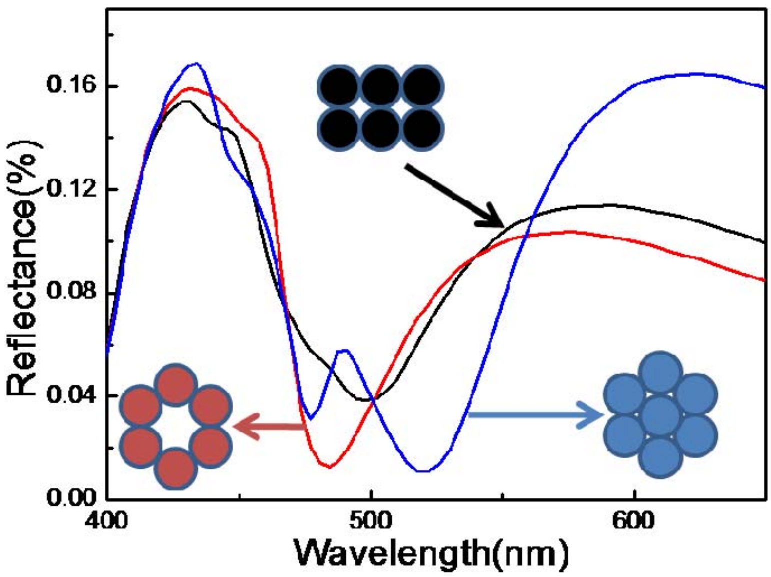

Supplementary Figure 6. MIT in some regular silicon nanosphere oligomers. The simulated reflectance spectra of some regular oligomers which had been widely studied in plasmonic structures. The diameter of silicon spheres was set to 100 nm.

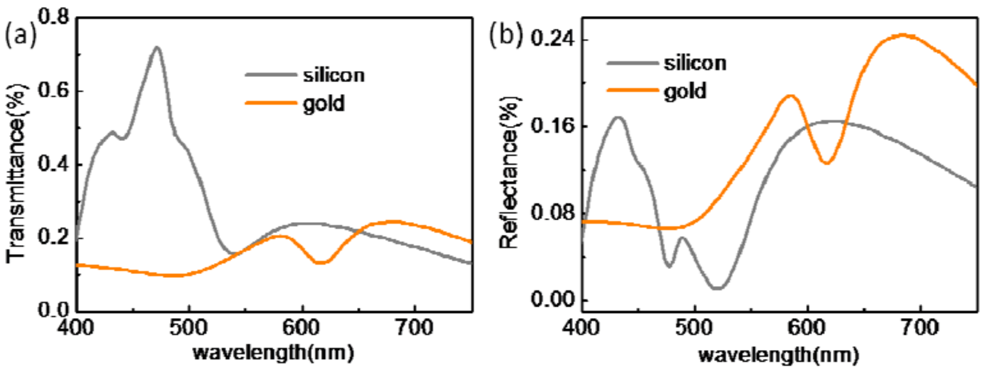

Supplementary Figure 7. MIT in some regular silicon nanosphere oligomers. (a) The transmittance spectra of silicon and gold heptamers. The diameters are set to 100 nm in both heptamers. (b) The reflectance spectra of silicon and gold heptamers.

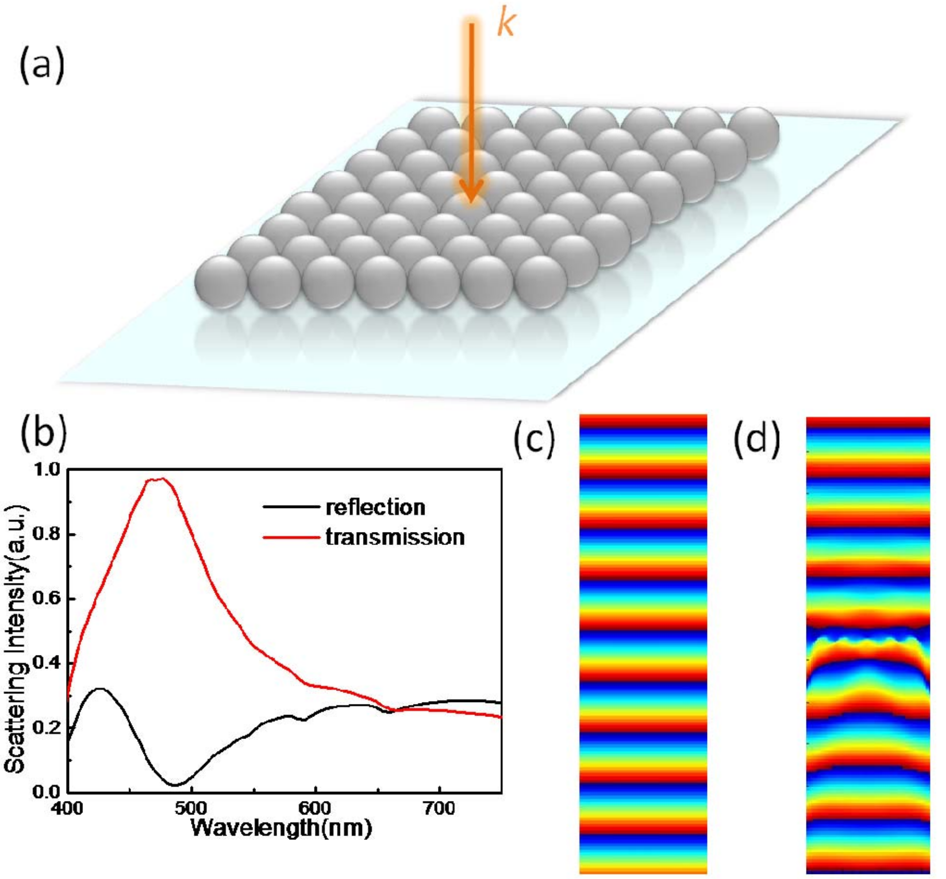

Supplementary Figure 8. Silicon nanosphere oligomers as epsilon-near-zero metamaterials. (a) The schematic diagram of silicon nanospheres array. Close-packed silicon nanospheres with diameters of 100 nm place on transparent substrate. (b) The simulated reflection and transmission spectra. (c) Phase change without silicon nanospheres array. (d) Phase change with silicon nanospheres array.
